# Review Global seroprevalence of legionellosis - a systematic review and meta-analysis

**DOI:** 10.1038/s41598-020-63740-y

**Published:** 2020-04-30

**Authors:** Frances F. Graham, Simon Hales, Paul S. White, Michael G. Baker

**Affiliations:** 1 Department of Public Health, University of Otago, Wellington, New Zealand; 2 Epidemiology and Laboratory Capacity Program, Commonwealth of the Northern Mariana Islands, Saipan, Commonwealth of the Northern Mariana Islands USA

**Keywords:** Bacterial infection, Epidemiology

## Abstract

*Legionella* is a ubiquitous pathogen yet the global occurrence of legionellosis is poorly understood. To address this deficit, this paper summarises the available evidence on the seroprevalence of *Legionella* antibodies and explores factors that may influence seroprevalence estimates. Through a systematic review, a total of 3979 studies were identified with seroprevalence results published after 1 January 1990. We tabulated findings by World Health Organization (WHO) region, location, study period and design, composition of study population(s) for all ages in terms of exposure, sex, detection methods, IFA titre, *Legionella* species measured, and present seroprevalence point estimates and 95% confidence intervals. Sampled populations were classified according to income, WHO region, gender, age, occupation and publication date. We conducted a meta-analysis on these subgroups using Comprehensive Meta-Analysis 3.0 software. Heterogeneity across studies was evaluated by the Q test in conjunction with *I*^2^ statistics. Publication bias was evaluated via funnel plot and Egger’s test. Fifty-seven studies met our inclusion criteria, giving an overall estimate of seroprevalence for *Legionella* of 13.7% (95% CI 11.3–16.5), but with substantial heterogeneity across studies.

## Introduction

Legionellosis is the collective term for the clinical syndromes caused by members of the genus *Legionella* that can present as either Legionnaires’ disease (LD) or Pontiac fever. Since the original description of the gram-negative bacterium in 1977^1^ more than 60 different *Legionella* species (spp.) have been described with over 70 serogroups^2^ with *L. pneumophila* serogroup 1 (sg1) the most prevalent disease causing variant^3^.

*Legionella* are largely environmental pathogens. Human-to-human transmission of *Legionella* may occur in rare cases^4,5^. There are no documented cases of zoonotic transmission^6^ despite *Legionella* antibodies being detected in the sera of animals^7–12^. The main threat of LD is from contaminated water (natural and artificial) systems colonised by the bacteria as well as natural soil and potting soil/compost^13^. Prolonged exposure of humans to environmental sources of *Legionella* triggers immune responses and the production of antibodies which are capable of persisting at measureable levels for several months and up to 10 years after exposure without causing any clinical symptoms^14^. Studies have shown that there is variation in *Legionella* antibody levels in healthy populations ranging from less than 1%^15^ to 45.1%^16^.

Most of our knowledge about the epidemiology of *Legionella* comes from testing patients who present with community-acquired or nosocomial pneumonia. The diagnosis is often missed because *Legionella* infection is difficult to distinguish from other forms of pneumonia, the unavailability of suitable testing or failure by clinicians to request it and the shortcomings of available diagnostic tests. Methods of diagnosing *Legionella* infections in clinical samples include culturing, antigen detection in urine, identification of the bacterium using paired serology, detection of the bacterium in tissue or body fluids by immunofluorescent microscopy, and genotypic polymerase chain reaction (PCR) methods^17^. Each method has its limitations, however serological methods for immunoglobulin M (IgM), G (IgG) and A (IgA) have an advantage in that they can determine whether or not a patient has had previous exposure to *Legionella*. Hence these methods have been described as an excellent technique to determine the seroprevalence of past and recent infection in a population^18^. The immunofluorescence assay (IFA) and the enzyme-linked immunosorbent assay (ELISA) are the two most widely used serological detection methods although the latter may appear to be less sensitive and specific when compared to IFA^19^. Microagglutination is also another method for serological diagnosis of legionellosis.

Epidemiological studies of *Legionella* have reported significant geographic variation in the seroprevalence of legionellosis both globally^20^ and domestically^21^. These studies have usually been cross-sectional and have almost always been used to determine levels of exposure in otherwise healthy populations or in different risk groups^22^. Generally, the prevalence of antibodies to *L. pneumophila* serogroup (sg) 1 has been reported since globally it is the species most frequently isolated. An Italian study showed significant diversity of antibody prevalence in different populations^23^. The prevalence of antibodies is not always strictly comparable due to the use of different diagnostic methods in laboratories and titre cut-off values. For example, a 4-fold or greater increase in reciprocal antibody titre to ⩾1:128 is considered a laboratory confirmed case of legionellosis^24^ while a single high titre of ⩾1:256, together with appropriate clinical features suggestive of legionellosis, is considered presumptive evidence of infection at an undetermined time. However, the latter definition should be used with caution since it has been shown that a single acute-phase antibody titre of ⩾1:256 could not discriminate between cases of clinical and sub-clinical disease^25^. In addition, the utility of serology which have low cut-off titre values can be complicated by cross-reactions which occur among *Legionella* spp. and other gram negative bacteria suggesting that serological cross-reaction is a common occurrence in routine *Legionella* serological testing both in patients with and without pneumonia^26,27^.

Despite several narrative reviews of the epidemiology of legionellosis^3,20,28,29^, to date there has been no systematic review or meta-analysis of published data that summarises the global seroprevalence of legionellosis (one review focussed on China^30^ and one on occupational risk^31^). Given the significant paucity of information, our aims were to 1) systematically search, assess and summarise the published work on the seroprevalence of *Legionella* globally and its epidemiology; 2) identify whether the seroprevalence data suggest an increasing risk of *Legionella* infection over time; 3) compare measured seroprevalence in ‘high-income’ versus ‘low-income’ countries; and 4) determine whether the prevalence of *Legionella* antibodies differed in ‘high risk’ occupations compared with ‘general populations’. Up-to-date epidemiological information is essential for planning public health interventions and identifying areas requiring further research.

## Materials and Methods

### Search strategy

We followed the PRISMA (Preferred Reporting Items for Systematic Reviews and Meta-Analyses) guidelines^32,33^ (refer to the PRISMA checklist outlined in Supplementary Fig. S2). We examined articles published from 1 January 1990 in Medline (Ovid), Embase, Scopus and the Cochrane Library. We deliberately included grey literature in our citation analysis search process via the following sources: Te Puna, Kiwi Research Information Service, Proquest Dissertations and Theses, Index to Theses, OCLC FirstSearch: WorldCat, EThOS (Electronic Theses Online Service), OAIster, DART-Europe E-Theses Portal, Theses Canada, Trove, as well as GreyLit.org and OpenGrey.eu. Figure S1 shows the search strategy. The main keywords used to identify potentially relevant studies included “legionellosis”, “legionella”, “Legionnaires disease”, “seroepidemiologic”, “prevalence” and “seroprevalence”. In circumstances where data were missing, we contacted the corresponding principal authors of the original studies. We also manually scrutinised the references citied by each potentially relevant paper to identify any additional eligible studies. Available grey literature was not considered useful for our review because it not contain original data on *Legionella* infection seroprevalence.

### Study selection

All study titles and abstracts obtained from the database searches were screened for eligibility by the principal author (FG). Suitable papers moved to the second stage where two reviewers independently assessed their eligibility according to the inclusion criteria. Legionellosis was defined as the pneumonic (LD) and non-pneumonic form (Pontiac fever) of infection caused by exposure to *Legionella* spp. In circumstances where multiple publications presented identical data sets and study period, only the most recent article was included. All languages were eligible for inclusion and no publication restrictions were applied. All non-English articles were screened using Google Translate^34^. Articles published after 1 January 1990 were selected only if an abstract contained data on the serological assessment of human samples for evidence of *Legionella* infection (LD and suspected Pontiac fever). To address the problem of varying thresholds, we included studies which reported IFA results where the serum samples were titrated from 1:64 and upwards to an end-point titre. To highlight the problem of different positivity thresholds used, all studies and their detection methods including reported titre cut-off to describe a positive antibody response to *Legionella* have been recorded in Supplementary Table S1. Studies which used the ELISA and rapid microagglutination tests to detect *Legionella*-specific antibodies were also included in our analysis. We excluded studies which (i) lacked a suitable denominator to assess seroprevalence, (ii) examined animal sera for *Legionella* antibodies, (iii) focused on *Legionella* spp. in the environment only, (iv) used IFA with a cut-off titre below 1:64 (although there is no definitive evidence that this is the optimal threshold)^35^, which were not considered meaningful due to false reactions and background staining^35^ and (v) analysed other pathogens in addition to *Legionella* using the same study populations which resulted in the inability to obtain specific *Legionella* data.

### Data extraction and statistical analysis

The following variables were extracted and tabulated: World Health Organization (WHO) region, location, study period, composition of study population(s) in terms of exposure, sex, detection method and IFA titre (upper limit considered positive) and *Legionella* spp. including serogroup that was measured (Supplementary Table S1).

For all qualifying studies, we extracted the number of subjects with antibodies against *Legionella* spp. and population size. To reduce heterogeneity for analysis, subgroup analyses were performed to assess the effect of geographic region (WHO), gender, occupation, age and publication year. Age was classified into three broad categories: children and adolescents ≤20 years; adults only (≥21 years) and all ages (children and adults combined). If a study did not state the population age range, it was included in the ‘all ages’ category. Countries were classified as high, middle or low income according to the World Bank data and thresholds for gross national income per person^36^.

The statistical analysis and graphical presentations were performed using the Comprehensive Meta-analysis (CMA) Version 3.0 software package developed by Biostat (Englewood,NJ) (http://www.meta-analysis.com) for comparing two groups with seroprevalence data. Seroprevalence rates were managed as a logit event estimate to normalize the distribution of data. Each logit event estimate was then transformed within the CMA software into proportions with 95% confidence intervals (CIs) when pooled analysis was undertaken. The overall seroprevalence rates were reported as percentages^37^. Data were assessed for heterogeneity using the Cochrane Q test, which has limited sensitivity, in conjunction with the *I*^2^ statistic, which represents the percentage of total variation across studies due to between-study heterogeneity^38^. The *I*^2^ was used to quantify inconsistency and values ≥75% were considered to represent a substantial degree of heterogeneity^39^. Where there was moderate to high between-study heterogeneity, a random-effects meta-analysis was used to produce pooled estimates for all outcome measures. To summarise the data visually and present 95% CIs, Forest plots were created. Publication bias was assessed using Egger’s test^40^ and funnel-plot-based methods as a means for assessing the validity of this meta-analysis.

No patient recruitment or other involvement in this study was required.

## Results

### Study selection

**Figure 1:**
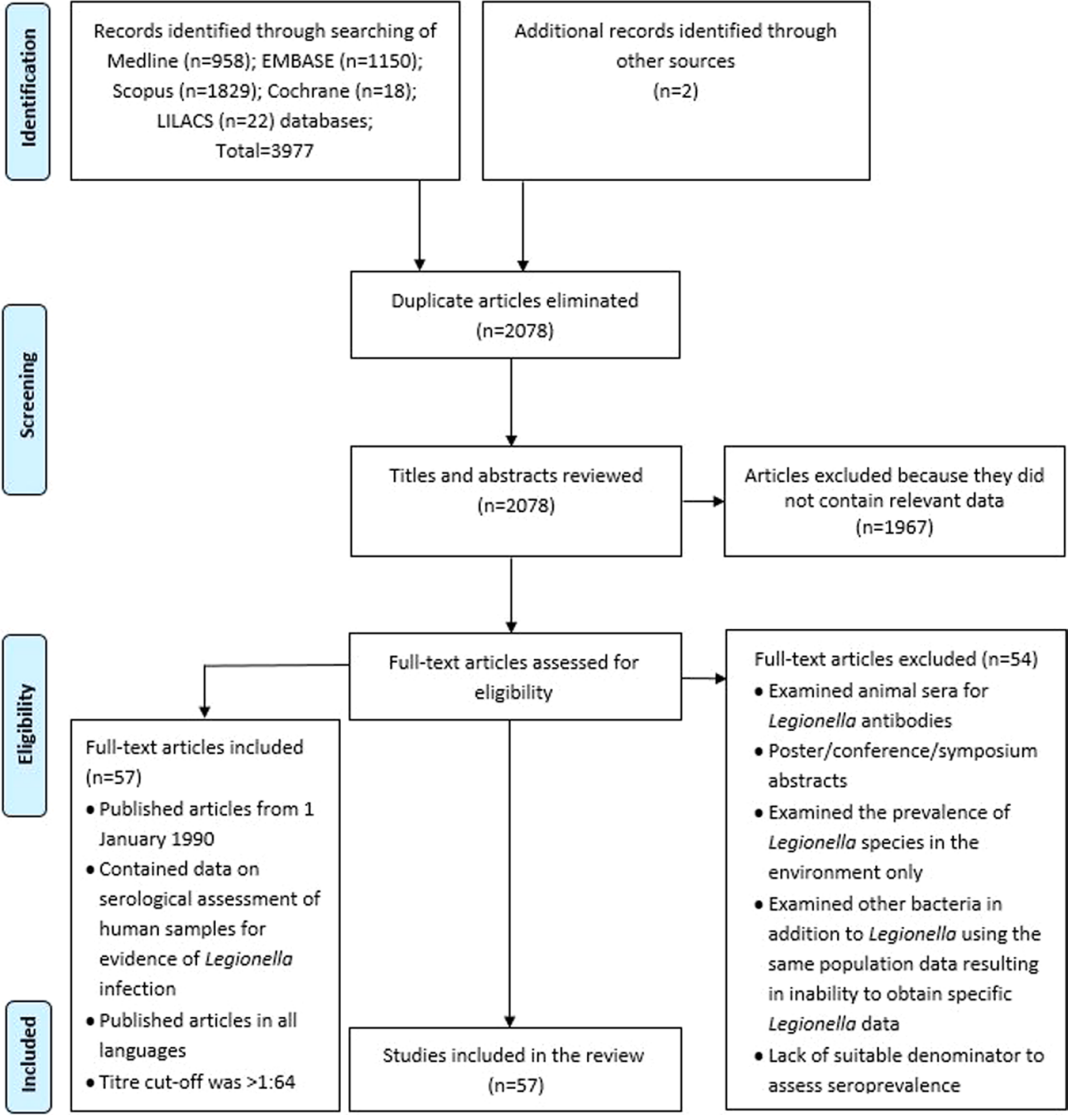
Results of the database searches and selection of eligible studies of *Legionella* seroprevalence.

Supplementary Fig. S1 summarizes the results of the search strategy. The literature search was completed on 30 June 2018. The search strategy retrieved 3977 unique citations; 958 were identified from MEDLINE, 1150 from EMBASE, 1829 from Scopus, 18 from Cochrane and 22 from LILACS. Of these 2078 citations were excluded based on duplicates after the first screening based on titles and abstracts, leaving 1967 to be examined (Fig. 1). After initial title and abstract review, 111 articles were read in detail after which 54 were excluded (Fig. 1). From these, we identified 57 articles that reported on the seroprevalence of LD in all ages of the general population (Fig. 1 and Supplementary Table S1).

### Characteristics of included studies

**Figure 2:**
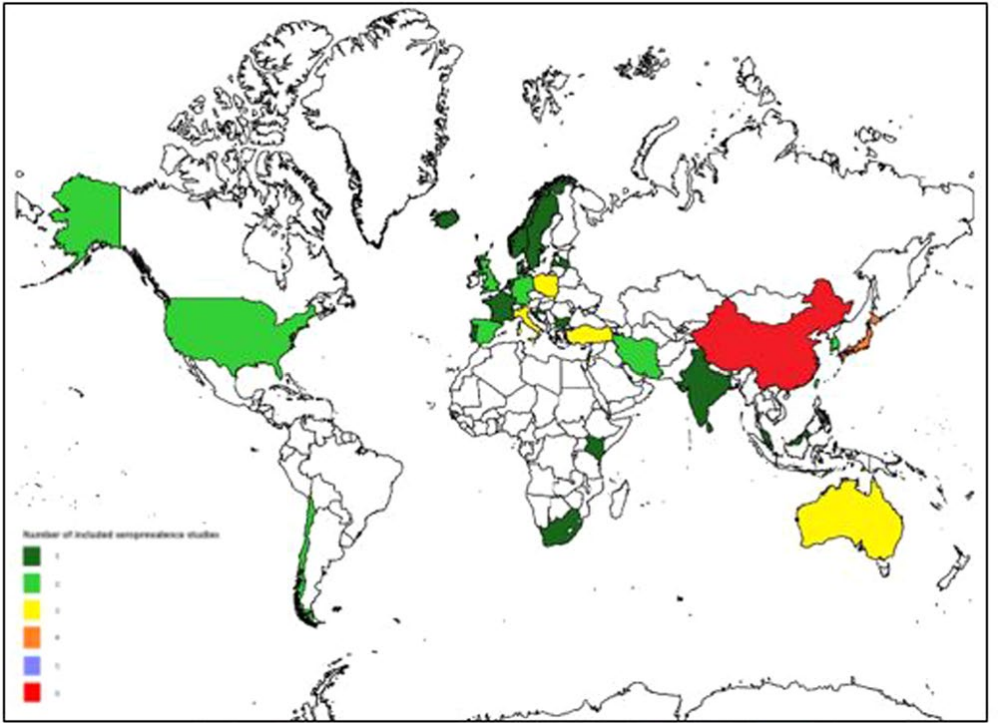
Map showing global distribution of the 57 included seroprevalence studies.

The sample size of these studies ranged from 25^41^ and 5431^42^ (median 252, interquartile range 122–604). Of the 57 studies, 53 were cross-sectional and 4 were cohort studies. IFA was used for laboratory screening in 32 of the 57 selected studies followed by ELISA (16) and microagglutination (9). Based on WHO geographic region, 26 studies were from Europe, 19 studies from the Western Pacific, 5 studies from the Americas, 3 studies from the Eastern Mediterranean and 2 each from South East Asian and Africa (Fig. 2).

### *Legionella* seroprevalence

**Figure 3:**
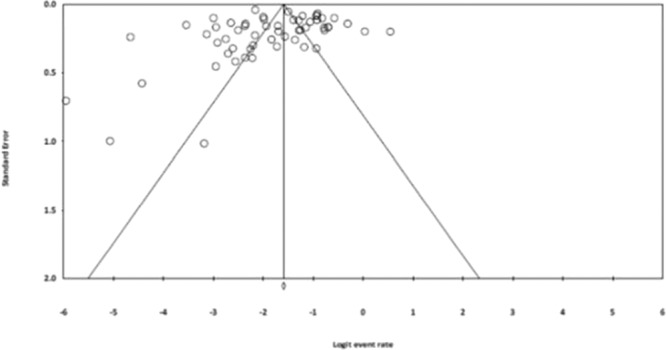
Forest plot of pooled seroprevalence of antibodies to *Legionella* (event rate) according to country status (high income verses low and middle income). Horizontal lines represent 95% confidence intervals (CIs). Each box represents the seroprevalence rate point estimate and its area is proportional to the weight of the study determined by inverse variance weighting. The *diamond* represents the overall summary estimate using the random effects model, with the 95% CI given by its width.

The overall random-effects pooled *Legionella* seroprevalence was 13.7 (95% CI: 11.3–16.5) with a high level of heterogeneity (*I*^2^ = 97.06%) (Fig. 3, Table 1). This analysis revealed significant heterogeneity across studies (p < 0.001). When only studies representing the general population (excluding occupational exposure) were considered, the pooled seroprevalence was decreased significantly to 10.5% (95% CI: 7.4–14.6) with still high heterogeneity (*I*^2^ = 96.52%) (Table 1) meaning that the seroprevalence differed when we excluded occupational exposure. The sensitivity analysis showed that regardless of which study was excluded, the results showed that no study had skewed the overall result. Studies reporting the prevalence of antibodies to *Legionella* in blood donors ranged from 1.2%^43^ to 41.7%^44^. The prevalence of antibodies to *L. pneumophila* sg 1 was reported in all studies with the exception of two serological investigations, one which found that the antibodies of non-*L. pneumophila* species such as *L. longbeachae*^45^, associated with exposure to compost and potting mixes^46^ may be highly prevalent in populations handling compost^44^. Another study of Icelandic children showed an absence to seroreactivity to *L. pneumophila* sg1 possibly due to antigenic and immunogenic differences between the strains used in the detection test^47^.

**Table 1:** Results of meta-analyses of the seroprevalence of antibodies to *Legionella* in total and by subgroup.

Meta-analyses/subgroup	Number of studies	Seroprevalence	*I*^2^
All studies	57	13.7% (95% CI: 11.3–16.5)	97.06
All studies (general population)	31	10.5% (95% CI: 7.4–14.6)	96.52
***Income (all studies)***			
High income	41	14.3% (95% CI: 11.4–17.9)	97.61
Middle income	15	13.3% (95% CI: 9.3–18.8)	93.05
Low income	1	1.2% (95% CI: 0.4–3.6)	0
***Income (general population)***			
High–income	24	10.9% (95% CI: 7.3–15.9)	97.11
Middle–income	6	13.7% (95% CI: 7.9–22.8)	85.26
Low–income	1	1.3% (95% CI: 0.4–4.1)	0
***WHO region***			
Africa	2	4.7% (95% CI: 0.3–41.6)	95.10
Eastern Mediterranean	3	12.0% (95% CI: 7.5–18.5)	70.61
European	26	14.7% (95% CI: 10.8–19.6)	97.32
South East Asian	2	12.4% (95% CI: 2.2–46.7)	71.03
The Americas	5	15.7% (95% CI: 6.9–31.7)	98.29
Western Pacific	19	13.0% (95% CI: 9.0–18.3)	97.11
***Gender***			
Male cases	7	7.0% (95% CI: 3.0–15.8)	95.23
Female cases	5	7.1% (95% CI: 2.7–17.5)	95.52
***Age***			
All ages	14	13.4% (95% CI: 9.2–19.3)	96.73
Adults only	40	13.5% (95% CI: 10.6–17.1)	97.32
Children/adolescents only (≤20 yrs)	3	15.9% (95% CI: 10.4–23.6)	78.39
***Occupation***			
Dentists	4	8.8% (95% CI: 3.9–18.7)	94.72
Healthcare workers (including aged care)	6	34.5% (95% CI: 21.9–40.5)	84.30
Commercial/Industrial workers	5	16.6% (95% CI: 5.6–39.7)	98.17
Drivers	2	3.7 (95% CI: 0.1–50.2)	90.12
Divers (professional)	1	28.3 (95% CI: 17.2–42.8)	0
Hotel workers	3	13.6 (95% CI: 4.6–33.7)	94.57
***Publication date***			
1990 to 1999	21	15.4% (95% CI: 11.9–19.7)	95.23
2000 to 2009	27	15.3% (95% CI: 11.6–19.8)	95.86
2010 to 2017	9	8.0% (95% CI: 4.6–13.7)	98.03

### *Legionella* seroprevalence for subgroups

The results of 6 meta-regression analyses for subgroups based on income, WHO region, gender, age, occupation and publication date are included in Table 1. There was an apparent higher seroprevalence in WHO regions such as Europe (14.7% (95% CI: 10.8–19.6)) and higher-income countries (14.3% (95% CI: 11.4–17.9)) possibly due to smaller numbers of studies from low to middle income countries making comparisons between other regions difficult.

Three studies reported the seroprevalence of LD in children and adolescents (defined as those aged ≤20 years) in Iceland, Asia and South America. The seroprevalence of *Legionella* amongst children and adolescents was 15.9% (95% CI: 10.4–23.6) which was higher than in adults 13.5% (95% CI: 10.6–17.1) and all ages combined (13.4% (95% CI:9.2–19.3)). The Taiwanese children’s study reported an increasing overall seroprevalence with age (10% in cases aged 12–18 months, increasing to 30% in the group aged 7–8 years; the seroprevalence showed a plateau from 9–18 years)^48^. In Chileans aged ≤20 years, seroprevalence was 10% (cut-off: ≥1:64) overall and 25% in higher socioeconomic groups^49^. There appeared to be little consistency within or between countries. For example, in Sweden, 0.2% of the general population had antibodies to *L. pneumophila* sg 1 five years after an outbreak, compared with 11% (IgG) and 16% (IgM) in Norway. The seroprevalence of LD in adults ranged from 0.2% to 43.4%. The examination of individuals of all ages yielded a higher seroprevalence of LD, 21.3% (95% CI: 20.1–22.6) and a range of 5.2% to 76.1%. The seroprevalence of *Legionella* was slightly higher among females (7.1%, 95% CI: 2.7–17.5) compared with males (7.0%, 95% CI: 3.0–15.8).

### Assessment of bias

**Figure 4:**
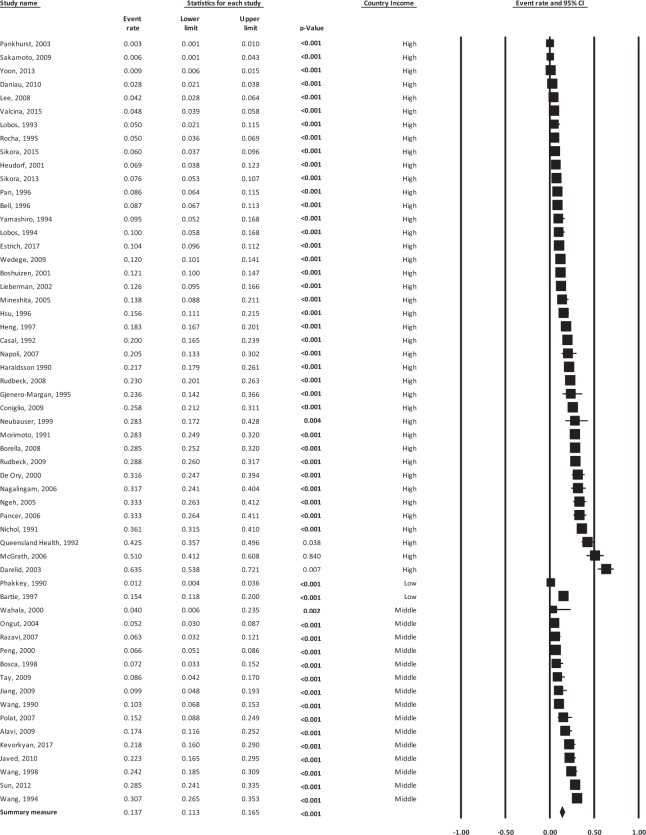
Funnel plot of standard error by logit effect size (event rate) for all studies (n = 57).

The funnel plot of standard error with logit effect size (event rate in this case) for all studies included in the meta-analysis did not identify significant publication bias (Fig. 4). Egger’s regression intercept tests (one-tailed) also revealed no evidence of publication bias (*ρ* = 0.13).

## Discussion

This systematic review provides the first published summary of the global epidemiology of legionellosis seroprevalence. Findings show that exposure to this organism is global in its distribution and common with an overall random-effects pooled seroprevalence for *Legionella* of 13.7% (95% CI 11.3–16.5). Seroprevalence for various sampled groups that met the inclusion criteria of this review varied widely from 0.2% to 76.1%. These variations reflected likely differences in exposure related to the type of population studied, location and season, as well as variations in testing methods (notably the screening test used, and antibody titre cut–off values).

Our findings did not identify evidence of increasing *Legionella* seroprevalence across the almost three decades covered by reported studies, though the number of studies was small. However, it is unknown to what degree the underlying seroprevalence of individuals correlates with national notification rates, since there is no globally accepted clinical case definition for Pontiac fever and LD^50^. For example, countries participating in the European Legionnaires’ Disease Surveillance Network (ELDSNet) only report cases with acute pneumonia (LD) in accordance with the 2012 EU/EEA case definition^51^. Nevertheless, as a result of global climate change, environmental conditions are likely to become increasingly favorable for the amplification of *Legionella* colonization in water systems particularly aging plumbing infrastructure, of urban areas^52^. Coupled with a growing predominantly urban population which is also aging population globally^53^, continuous human consumption of drinking water from aging infrastructure and the increased use of artificial water systems to deliver air conditioning, could result in high absolute seroprevalence in parallel with higher relative risk to human health. This hypothesis is consistent with literature demonstrating a higher risk of legionellosis acquisition in urban areas compared to rural regions^54^ due to increased exposure to artificial water systems such as cooling towers for air conditioning and more collective hot water systems^54–56^.

Previous outbreak studies have detected elevated antibody levels among individuals exposed to *L. pneumophila*, and although these individuals did not develop overt LD the evidence might suggest a degree of past non-clinical exposure. Given that many widespread and diverse water systems and non-water systems are reservoirs of *Legionella* and many diverse systems and matrices have been reported as sources of *Legionella*^13^, it is possible that individual differences in behaviour and risk factors could account for varying seroprevalence of antibodies to one or more *Legionella* spp. in the population. Risk factors associated with the occurrence of legionellosis are not fully understood but some studies have suggested that genetic factors may enhance susceptibility to LD^57^. Legionellosis varies by age although the importance of *Legionella* spp. should be considered in all age groups^58^ including children^59–62^. Of interest were two of the three studies which reported the seroprevalence in children and adolescents both used the same *Legionella* IFA Kit (Organon Teknika, USA) to detect antibodies to *L. pneumophila* sg1–6. However, the potential contribution arising from antibody cross reactivity to other Gram-negative bacterial antigens was not investigated by the study authors^47,48^. Seroprevalence in males generally exceeds that in females although there are exceptions^54^. Other risk factors for the disease include tobacco smoking^63^ and pre-existing conditions such as liver cirrhosis^64^, chronic obstructive pulmonary disease, cancer, diabetes mellitus and immunosuppression.

High socioeconomic factors were associated with a raised prevalence of *Legionella* antibodies in Chile^49^. One study which evaluated demographic characteristics such as race/ethnicity reported the highest seroprevalence of LD among the white population^42^. Despite being the inverse to the usual male/female ratio trend, seroprevalence was observed to be slightly higher in females (7.1%) than males (7.0%) which is consistent with a recent study^65^ although a plausible explanation could most likely be sought in the low number of studies that were eligible for our review. Nevertheless, one study has shown that women could be more resistant to LD due to the role of Toll-like receptor polymorphisms which protect from an infection^66^.

Cases of LD in occupational settings are widely reported and workers in specific professions with exposure to aerosols may be at higher risk for the disease^62,67^. Our results showed that the overall pooled *Legionella* seroprevalence across the studies was 13.7% but decreased to 10.5% when occupation exposure was excluded (Table 1). Occupational subgroup analysis in this study showed that some occupations seemed to be at higher risk of antibody response to *L. pneumophila*, namely car and bus drivers^68,69^, professional divers^70^, dental^15,42,71,72^, hospital^16,73^ and hotel staff^74^ and workers from industrial/commercial settings^75–78^. *Legionella* antibody titres in the blood of dental workers were higher than in the overall population, suggesting that aerosols generated by dental unit waterlines instruments were the primary source^79^. This finding may be a reflection of the rich microbial biofilms commonly present along the length of the fine-bore dental water hoses which contributed to the heavy contamination^80,81^. Nevertheless another study found that the overall prevalence of *L. pneumophila* antibodies was lower (approximately 10%) and did not significantly vary between those who were involved in the delivery of dental care and those who were not^42^. Such a contrast may be the result of the United States Centers for Disease Control and Prevention (CDC) in 1993 releasing infection control guidelines in dental healthcare settings at a time when there was a higher risk of *Legionella* infection^31^. Despite the low observed seroprevalence in a population comprising of nuclear power plant workers exposed to aerosol-generating sources via cooling towers Daniau *et al*., showed that for exposure from *L. pneumophila* sources not wearing a mask for respiratory protection was a significant risk factor for positive *Legionella* results^75^. Other studies which focused on non-*L. pneumophila* species showed high antibody positivity to *L. longbeachae* in potting media industry workers^44^. This corroborates the notion that cases of *L. longbeachae* infection are frequently associated with exposure to potting mix/soils and composts^46,82,83^.

Our meta-analysis identified some geographic variation in legionellosis, but it is based on limited numbers of studies from most regions. Legionellosis is a ubiquitous complex disease that is influenced by a variety of natural and artificial factors (which can promote its proliferation to high concentrations)^84^ environmental factors as well as withstand a wide range of temperatures (<0 °C to 60 °C)^85^. Seroprevalence for various sampled groups that met the inclusion criteria of this review varied from 0.2% to 76.1%. Variations depended on the type of population studied, location, season, detection method used and antibody titre cut-off value (Supplementary Table S1). For example, an Italian multicentre study showed seroprevalence against *L. pneumophila* sg 1–6 (Naples) was 3.4% compared to 16.4% against *L. pneumophila* sg 7–14 (Milan). The main factors underlying the observed differences was due to the detection and/or reporting cases, and diverse age composition of the two populations (healthcare workers and blood donors)^86^. The spatial disparities encountered, however, did not suggest that variation in seroprevalence of legionellosis depended on the distance from the equator.

Continuous environmental exposure of humans to the bacteria from *Legionella*-contaminated sources may stimulate immune responses and generate antibodies^54^. Sero-surveys amongst participants in an outbreak investigation showed that exposure to the bacteria causes increased antibody levels in individuals who do not develop LD and that this effect was higher for those closest to the source^87^. Our findings also assessed health outcomes of *Legionella* infection in highly exposed populations beyond the outbreak situation^86,88^. In HIV-infected patients, no association was proven with the investigated risk factors for legionellosis, the difference in seroprevalence to *Legionella* spp. and serogroups dependent on their immune status. Immune responses namely that antibodies to less virulent *L*. *pneumophila* sg7–14 and non-*pneumophila* are less systematically manufactured in HIV infected patients, compared to more virulent *L. pneumophila* sg1–6 that are capable of better arousing the immune system have been hypothesised^89^. Antibody response was not associated with other immunosuppressive disorders such as chronic renal failure (hemodialysis patients)^90^ and post-renal transplantation^91^. In another study, Morimoto concluded that the titre in hemodialysis patients was higher than the control group (p < 0.005)^92^. The frequency of antibodies to *L. pneumophila* in patients with autoimmune rheumatic diseases was comparable to that in healthy individual patients with this disease being more susceptible to infection owing to the underlying disease itself, comorbidities or to its treatment namely the use of immunosuppressants (including anti-TNF-α)^93^. On the other hand legionellosis may be more prevalence among patients hospitalized for acute exacerbations of chronic obstructive pulmonary disease to account for the clinical expression of exacerbations in these patients being characterised by gradual onset and increasing systematic manifestations^94^. Hence, such patients should be appraised with priority, including diverse populations likely to be more at risk^95^.

Studies of *Legionella* seroprevalence have important limitations which in turn limit the conclusions of our meta-analysis. Firstly, while the optimal time for detecting antibodies is generally within a few weeks after onset of the disease^14^, high levels of antibodies can persist for years after the infection^14^ making interpretation of elevated titres difficult. This means that seroprevalence cannot be interpreted as either a measure of recent infection (incidence) nor as a measure of long-term exposure risk (cumulative incidence). Secondly, interpretation of the seroprevalence will not always be strictly comparable because of a lack of a standardized approach between laboratories in their methods employed to detect antibodies to *Legionella* spp^75^. and titre cut-off values. We found many studies employed different cut-off titre values to define seropositivity meaning that a simple review of results could be misleading. Of significance is the use of a diverse range of in-house and commercially manufactured IFA and enzyme immunoassay antigen preparations which may complicate the interpretation of antibody titres for *Legionella*, in particular over time and from different studies^96^. For example, in European countries such as Denmark, positive serology rates are systematically confirmed by national reference centers that perform in-house techniques due to a lack of specificity of commercial kits developed for the detection of antibodies to *Legionella*^16,97^. Lastly, seroprevalence studies are not a good indicator as to the severity or type of infection namely subclinical, non-pneumonic disease (Pontiac fever), LD or extra pulmonary disease^98^. The impact of this is that while once popular for LD diagnosis, globally the trend is that the scope and number of serological tests performed in the laboratory setting is dropping significantly due to the increase in standardized techniques and culture media in addition to faster, more definitive analyses such as the rapid urinary antigen test and molecular methods. This observation was reflected in our results which showed a significant drop off in the number of published studies between 1990 and 2010 particularly in high-income countries. For example, in Europe the use of serology for LD confirmation decreased from 61% to 6% on average in the period from 1995 to 2010 in favour of rapid, less technically demanding urine antigen test or molecular diagnostic tools^96^. Acknowledging these limitations, serological diagnostic tests used in epidemiological investigations can provide useful retrospective data on the cumulative incidence of the disease^96^ as well as potential recurrent outbreaks, since it is the only means of assessing the number of undiagnosed cases.

To conclude, we present a systematic review and meta-analysis of seroprevalence studies of *Legionella* infection to gain a better understanding of the global distribution of this disease. We acknowledge significant heterogeneity was found when data were pooled due to different characteristics among identified studies despite using a random-effects model to provide a more conservative result so the outcome of this pooling needs to be interpreted with caution. For example, the studies that we included were primarily in urban areas where *Legionella* is endemic. Nevertheless, we believe our meta-analysis provides the most comprehensive description of the global seroprevalence of *Legionella* so far published. Given that most studies identified in this review were cross-sectional (53 of 57) further cohort and case-control studies of non-outbreak disease are needed to expand our knowledge of risk factors and exposures for this disease.

## Supplementary information

Supplementary information.
