# Supplementary material for: Review Global seroprevalence of legionellosis - a systematic review and meta-analysis
Source: Sci Rep. 2020 Apr 30;10:7337. doi: 10.1038/s41598-020-63740-y (PMC7193644; doi:10.1038/s41598-020-63740-y)
Supplement: Supplementary file 1 — Supplementary information. [file 41598_2020_63740_MOESM1_ESM.docx]

**Supplementary Information**

**Global Seroprevalence of Legionellosis – a Systematic Review and Meta-analysis**

**Frances F Graham,^a^ Simon Hales,^a^ Paul S White,^b^ and Michael G Baker^a^**

**a** Department of Public Health, University of Otago, Wellington, New Zealand

**b** Epidemiology and Laboratory Capacity Program, Commonwealth of the Northern Mariana Islands, Saipan

Database: Ovid MEDLINE(R) In-Process & Other Non-Indexed Citations and Ovid MEDLINE(R) <1946 to Present>, adapted for Embase, Scopus, Cochrane Library, LILACS

Search Strategy:

--------------------------------------------------------------------------------

1 exp legionella/

2 legionell*.ab,ti.

3 legionnaire*.ab,ti.

4 legionellosis/

5 legionnaire's disease/

6 1 or 2 or 3 or 4 or 5

7 prevalence/

8 (prevalence or "prevalent stud*" or "antibod*" or "cross-sectional stud*").ab,ti.

9 seroepidemiologic studies/

10 seroprevalence.ab,ti.

11 7 or 8 or 9 or 10

12 6 and 11

13 limit 12 to yr="1990 -Current"

**Figure S1.** Search strategy for seroprevalence and *Legionella*

1. Title - The report is identified as a systematic review with a meta-analysis

2. Structure summary - The structured abstract includes objectives, data sources, study selection, data extraction, data synthesis and conclusions.

3. Rationale - Described in the Introduction

4. Objectives - Stated in the Introduction

5. Protocol and Registration – The protocol is described in the Methods and in Figure S1 and conforms to the Preferred Reporting Items for Systematic reviews and Meta-analyses (PRISMA-P 2015) guidelines. Registration does not apply

6. Eligibility criteria – They are defined in the Methods

7. Information sources – Described in the Methods

8. Search – Described in the Methods and Figure S1

9. Study selection - Described in the Methods

10. Data collection process – Described in the Methods

11. Data items – Described in Methods and summarized in Table S1

12. Risk of bias in individual studies –We explored heterogeneity with a variety of methods (Methods and Table 1), including quality of study.

13. Summary measures – 95% Confidence Intervals

14. Planned methods of analysis – Described in Statistical Analysis

15. Risk of bias across studies – Described in Statistical Analysis and reported in detail in Results

16. Additional analyses – Sensitivity analysis, sub-group analysis (see Figure 3 and Table 1)

17. Study selection – see Flow diagram outlined in Figure 1

18. Study characteristics – shown in Table S1

19. Risk of bias within studies – see 12

20. Results of individual studies – shown in Forest plot

21. Syntheses of results – shown in Results and in Forest plot caption

22. Risk of bias across studies – see 15

23. Additional analyses – results reported in Table S1

24. Summary of evidence – stated early in Discussion

25. Limitations – addressed in Discussion

26. Conclusion – addressed in the last paragraphs of manuscript

27. Funding – not applicable in this instance

**Figure S2**. The PRIMSA checklist†

† Liberati A, Altman DG, Tetzlaff J, *et al*. The PRISMA statement for reporting systematic reviews and meta-analyses of studies that evaluate healthcare interventions: explanation and elaboration. *Br Med J.* **339,** b2700 (2009).

**Table S1.** Description of characteristics of the seroprevalence studies published from 1 January 1990 to determine anti-*Legionella* antibodies according to WHO region

| **No.** | **Study Reference** | **Study Characteristics** | | | | | | | |
| --- | --- | --- | --- | --- | --- | --- | --- | --- | --- |
|  | **First Author Year Ref** | **WHO Region** | **City or Region/Country** | **Study period (for sample collection)** | **Groups sampled** | **Sex**  **(no. positives)** | | **Detection methods and titre cut-off (described in article as positive)** | ***Legionella* species and serogroup (sg) measured** |
|  |  |  |  |  |  | **M** | **F** |  |  |
| 1 | Borella, 2008 [1] | European | Northern (Milan and Turin) and Southern (Naples and Bari)/Italy | NR* | Healthcare workers (a)  Students (medical & dental) (b)  Blood donors (c) | 131 | 209 | IFA to detect IgM and IgG ((IFA, RIDA FLUORlegionella IgG, R-Biopharm AG, Darmstadt, Germany); ≥1:256 | *L. pneumophila* sg1–14 |
|  |  |  |  |  |  | NR* | NR* |  |  |
|  |  |  |  |  |  | NR* | NR* |  |  |
| 2 | Bosca, 1998 [2] | European | Ankara/Turkey | NR* | Hospital patients (a)  Control group (healthy volunteers) (b) | 27  (2) | 26  (4) | IFA to detect IgM, IgG and IgA  ≥1:256 (a)  ≥1:128 (b) | *L. pneumophila* sg1–6 |
|  |  |  |  |  |  | NR* | NR* |  |  |
| 3 | Boshuizen, 2001 [3] | European | Bovenkarspel/The Netherlands | Mar 1999 | Exhibitors at a floral show | 423 | 369 | ELISA to detect IgM and IgG using the commercial kit (Serion classic ELISA; Virion-Serion) | *L. pneumophila* sg1–7 |
| 4 | Casal, 1992 [4] | European | Cordoba/Spain | Jun 1986-Aug 1989 | Hospital (mainly pulmonary) patients | 304 | 147 | IFA ((BioDx and SciMedx kits); ≥1:64 | *L. pneumophila* sg1–6 |
| 5 | Coniglio, 2009 [5] | European | Sicily, Italy | Sep-Oct 2004 | HIV-infected subjects (a)  Blood donors (b) | 78 | 25 | IFA to detect IgM and IgG (using IFA, RIDA FLUORlegionella IgG, R-Biopharm AG, Darmstadt, Germany); ≥1:128 | *L. pneumophila* sg1–6  *L. pneumophila* sg7–14 |
|  |  |  |  |  |  | 143 | 52 |  |  |
| 6 | Daniau, 2010 [6] | European | France | Jan-Dec 2002 | Industrial workers | 1387 | 73 | ELISA to detect IgM and IgG (using SERION ELISA classic: ref kit ESR106G and ESR106M); IFA for IgG (using Meridan Diagnoses Europe); ≥1:128) | *L. pneumophila* sg1–7 |
| 7 | Darelid, 2003 [7] | European | Jonkoping County/Sweden | 1991 -2001 | Hospital patients (a)  Reference population (b) | NR* | | IFA to detect IgM; ≥1:128 (CDC criteria) | *L. pneumophila* sg1 |
| 8 | De Ory, 2000 [8] | European | Madrid/Spain | Aug-Sep 1996 | Patients from a community outbreak (confirmed cases) | NR* | | IFA to detect IgM; ≥1:128 | *L. pneumophila* sg1 |
| 9 | Gjenero-Margan, 1995 [9] | European | Split/Croatia | Jan 1991-Feb 1994 | *Pityriasis rosea* patients (a)  Blood donors (b) | NR* | | IFA using the commercial Zeus Technologies Inc. | *L. pneumophila* sg1–6  *L. micdadei* |
| 10 | Haraldsson, 1990 [10] | European | Reykjavik/Iceland | Jun-Dec 1985 | Children (≥ 1mon to ≤12 years) with acute lower respiratory tract infections | 216  (34 - ≥1:64 only) | 180  (52 -≥1:64 only) | Microagglutination test to detect IgM antibodies | *L.pneumophila* sg1–6  *L. micdadei*  *L. bozmanae*  *L. dumoffii* |
| 11 | Heudorf, 2001 [11] | European | Frankfurt/Germany | 1997-1998 | Inhabitants with central hot-water systems (a)  Inhabitants with de-centralised hot water systems (control) (b) | 15 | 38 | ELISA to detect IgG and IgM (Virion Serion GmbH, Würzburg, Germany); ≥1:128 | *L.pneumophila*  *L. micdadei*  *L. bozmanae*  *L. jordanis*  *L. longbeachae*  *L. dumoffii* |
|  |  |  |  |  |  | 45 | 47 |  |  |
| 12 | Kevorkyan, 2017 [12] | European | Plovdiv province/Bulgaria | May-Nov 2015 | Healthcare (medical/dental) personnel (a)  Control group (b) | 12  (5) | 54  (22) | ELISA to detect IgM and IgG (Virell, Spain; (serum antibody index >5) | *L. pneumophila* sg1–6 |
|  |  |  |  |  |  | 44  (2) | 46  (5) |  |  |
| 13 | Napoli, 2007 [13] | European | Bari/Italy | NR* | Dental professionals (a)  Controls (b) | 20 | 24 | IFA (RIDA-Fluor-Legionella IgG-rBiopharm); ≥1:128 | *L. pneumophila* |
|  |  |  |  |  |  | 19 | 25 |  |  |
| 14 | Neubauer, 1999 [14] | European | Eckernförde/Germany | NR* | Divers (a)  Non-diver controls (b) | NR* | | IFA for IgM and IgG; ≥1:64 | *L. pneumophila* |
| 15 | Ngeh, 2005 [15] | European | London, England | Dec 1999-Mar 2000 | Elderly patients with stroke or transient ischemic attack (a)  Controls (no past history of a stroke) (b) | NR* | | ELISA kit (Vircell SL) was used for the analysis of IgG and IgM  antibodies in serum | *L. pneumophila* sg1 |
| 16 | Ongut, 2004 [16] | European | Turkey | Sep-Nov 2002 | Hemodialysis patients | NR* | | ELISA to detect IgM, IgG and IgA (Zeus Scientific, Inc, Raritan, NJ, USA) | *L. pneumophila* sg1–6 |
| 17 | Pancer, 2006 [17] | European | Warsaw/Poland | NR* | Hospital workers (a)  Blood donors (b) | NR* | | Microagglutination test; ≥1:64 | *L. pneumophila* sg1–15  *L. micdadei*  *L, bozemanae*  *L. jordanis*  *L. anisa* |
| 18 | Pankhurst, 2003 [18] | European | London/England and Northern Ireland | NR* | Dentists (a)  Blood donors (b) | 224 | 22 | IFA; Dentist 1:128 (a)  Blood donor 1:64 (b)  (range ≤1:16 -1:128) | *L. pneumophila* sg1–6 and 8 |
|  |  |  |  |  |  | NR* | NR* |  |  |
| 19 | Polat, 2007 [19] | European | Denizii/Turkey | Feb-Aug 2004 | Bus drivers (a)  Driver assistances (control) (b) | 63  (12)  16  (0) | 0  0 | IFA (LpSg1-14, Euroimmun, Biochip Sequence, Germany) | *L. pneumophila* sg1–14 |
| 20 | Rocha, 1995 [20] | European | Northern Portugal | May-Nov (year not stipulated) | Patients (a)  Therapists (b)  Blood donors (c) | 49 | 123 | IFA (using antisera (obtained from  the Public Health Laboratory Service, London, UK) to LpSg1-13); ≥1:256  (≥1:64-≥1:128 considered borderline for Lp; ≥1:128 for other *Legionella* spp.) | *L. pneumophila* sg1–10  *L. bozmanae* sg1–2  *L. londiniensis* |
|  |  |  |  |  |  | 12 | 30 |  |  |
|  |  |  |  |  |  | NR* | NR* |  |  |
| 21 | Rudbeck, 2008 [21]; 2009 [22] | European | Randers and Vejle/Denmark | Feb-Jun 2004 | Blood donors (Randers) (a)  Blood donors (Vejle) (control) (b) | 403 | 305 | IFA test; ≥1:128 | *L. pneumophila* sg1 |
| 22 | Rudbeck, 2009 [23] | European | Denmark  (NB Randers and Vejle, blood donors only) | Sep-Nov 2005 | Hospital workers (a)  Blood donors (b) | 36 | 222 | In-house IFA test; ≥1:128 | *L. pneumophila* |
|  |  |  |  |  |  | 403 | 305 |  |  |
| 23 | Sikora, 2015 [24] | European | Poland | NR* | Patients with autoimmune rheumatic disease (a)  Healthy blood donors (b) | 48 | 117 | ELISA to detect IgM and IgG using two commercial kits (EUROIMMUN Medizinische Labordiagnostika AG, Germany; Vircell, Spain) | *L. pneumophila* sg1–7 |
|  |  |  |  |  |  | 28 | 72 |  |  |
| 24 | Sikora, 2013 [25] | European | Lublin/Poland | Nov 2010-Mar 2011 | Dialysis patients (a)  Patients post renal transplantation (b)  Healthy blood donors (volunteers) (c) | NR* | NR* | ELISA to detect IgM, IgG using two commercial kits (EUROIMMUN Medizinische Labordiagnostika AG, Germany; Vircell, Spain) | *L. pneumophila* sg1–7 |
|  |  |  |  |  |  | NR* | NR* |  |  |
|  |  |  |  |  |  | 72 | 28 |  |  |
| 25 | Valcina, 2015 [26] | European | Latvia | Feb-Oct 2014 | Blood donors | 886 (29) | 1121 (67) | IFA to detect IgM and IgG for LpSg1-6 using Vicell, Spain; ELISA IgG, Vircell, Spain for LpSg1 for all positive samples | *L. pneumophila* sg1–6 |
| 26 | Wedege, 2009 [27] | European | Østfold/Norway | 2006 | Factory workers (a)  Exposed blood donors (b)  Non-exposed blood donors (c) | 192 | 21 | ELISA (IgG and IgM considered positive if antibody index >11) | *L. pneumophila* sg1–7 |
|  |  |  |  |  |  | 188 | 210 |  |  |
|  |  |  |  |  |  | 226 | 180 |  |  |
| 27 | Alavi, 2009 [28] | Eastern Mediterranean | Ahvaz/Iran | Dec 2007-Nov 2008 | Hospital patients | 71 (14) | 50  (7) | ELISA (Vircell, Spain) to detect IgG and IgM | *L. pneumophila* sg1–6 |
| 28 | Lieberman, 2002 [29] | Eastern Mediterranean | Beer-Sheva/Israel | Nov 1997-Mar 1999 | Patients with exacerbation of chronic obstructive pulmonary disease (a)  Control group (b) | NR* | | IFA to detect IgM and IgG using in-house kit; ≥1:64 | *L. pneumophila*  *L.bozmanae*  *L. feeleii*  *L. jordanis*  *L. oakridgensis*  *L.gormanii*  *L.micdadei*  *L. erythra*  *L. nautarum*  *L gratiana*  *L. londinensis* |
| 29 | Razavi, 2007 [30] | Eastern Mediterranean | Tehran/Iran | 2004-2005 | Volunteer Iranian Hajj pilgrims (before and after the pilgrimage) | 143 | 27 | ELISA; ≥1:64 | *L. pneumophila* |
| 30 | Bartie, 1997 [31] | Africa | Kwazulu-Natal/South Africa | Aug 1991-Oct 1992 | Mine workers (a)  Factory workers (b) | 155  (41) | 0 | IFA (Zeus Scientific, New Jersey, USA); ≥1:64 | *L. pneumophila* sg1–4 |
|  |  |  |  |  |  | 143  (5) | 0 |  |  |
| 31 | Phakkey, 1990 [32] | Africa | Nairobi/Kenya | NR* | Hospital patients with a diagnosis of pneumonia (a)  Blood donors (b) | NR* | | Microagglutination test; ≥1:64 | *L. pneumophila* sg5  *L. bozmanae* |
| 32 | Bell, 1996 [33] | Western Pacific | Sydney/Australia | Apr-Jun 1993 | Seminar attendees at hotel (a)  Randomly selected population (b) | 92 | 60 | IFA; ≥1:128 | *L. pneumophila* sg1 |
|  |  |  |  |  |  | NR* | NR* |  |  |
| 33 | Heng, 1997 [34] | Western Pacific | Singapore | 1992-1995 | Residential population (<20yrs) (a)  Residential population (≥20yrs) (b)  Occupational groups (c)  Occupants with building related illness (d) | NR* | | IFA using polyvalent and monovalent DFA conjugates from MarDx Diagnostics, USA, DFA monoclonal antibody reagent (Sanofi Diagnostics, Pasteur, France), and slide agglutination using the Denka Seiken Kit, Japan; ≥1:256 | *L. pneumophila* |
| 34 | Hsu, 1996 [35] | Western Pacific | Taiwan | 1994-1995 | Children (7-18 yrs) (a)  Children (control) (12-18 mths) (b) | NR* | | IFA commercial kit (Organon Teknika, USA) (7-18 yrs) 1:64-1:2058 (27) children (control) (12-18mths) 1:64 (2) (range ≤1:16-1:2048) | *L. pneumophila* sg1–6 |
| 35 | Jiang, 2009 [36] | Western Pacific | Dalian/China | Aug-Sep 2005 | Workers in public places | NR* | | Microagglutination test | *L. pneumophila* sg7–10 |
| 36 | Lee, 2008 [37] | Western Pacific | Jeollanam-do/South Korea | Jan-Jul 2004 | Residential population | 242  (6) | 258  (15) | IFA for the detection of IgM and IgG; ≥1:128 | *L. pneumophila*  *L. bozemanae* |
| 37 | McGrath, 2006 [38] | Western Pacific | Yamanto (Queensland)/ Australia | Oct-Nov 2005 | Yamanto Police Station employees (a)  Workplace Health and Safety Queensland (control group) (b) | NR* | | IFA to detect IgG using a commercial testing kit (MarDx Diagnostics Inc, USA); ≥1:64 | *L. pneumophila* sg1–6  *L. longbeachae* |
| 38 | Mineshita, 2005 [39] | Western Pacific | Japan | Nov 1998 | Inpatients of nursing home (a)  Staff of nursing home (b) | NR* | | Microagglutination test; ≥1:128 | *L. pneumophila* sg5 |
| 39 | Morimoto, 1991 [40] | Western Pacific | Tokyo/Japan | NR* | Hemodialysis patients (a)  Controls (b) | NR* | NR* | IFA | *L. pneumophila* sg1–6 |
|  |  |  |  |  |  | 235 | 118 |  |  |
| 40 | Pan, 1996 [41] | Western Pacific | Taiwan | NR* | Hospital patients | 310  (32) | 177  (10) | Microagglutination test | *L. pneumophila* |
| 41 | Peng, 2000 [42] | Western Pacific | Beijing/China | 1997-1999 | Hotel workers (a)  Residential population (b) | NR* | | Microagglutination test | *L. pneumophila* sg1–16 |
| 42 | Queensland Health, 1992 [43] | Western Pacific | Brisbane/Australia | 1992 | Potting mix industry workers (a)  Blood donors (b) | NR* | | IFA; ≥1:64 | *L. longbeachae* |
| 43 | Sakamoto, 2009 [44] | Western Pacific | Japan | NR* | Employees of regional companies  (e.g. transport, construction) | 159 | 0 | ELISA to detect IgG and IgM (considered positive if antibody index >11 at a dilution of ≥1:20) | *L. pneumophila* sg1–6 |
| 44 | Sun, 2012 [45] | Western Pacific | Changzhou/China | Oct 2008 | Hotel workers exposed to contaminated cooling towers (a)  Control group (b) | 59 | 139 | Commercial IFA testing kits (Biochip Technology – Euroimmun, Lübeck, Germany); ≥1:100 | *L. pneumophila* sg1–14 |
|  |  |  |  |  |  | 51 | 105 |  |  |
| 45 | Tay, 2009 [46] | Western Pacific | Kuala Lumpur/Malaysia | 2006-2007 | Hospital patients with respiratory illness (a)  Blood donors (b) | NR* | | ELISA to detect IgM/IgG and IFA to detect IgM using testing kits - Vircell, Spain (≥1:96) | *L. pneumophila* sg1 |
| 46 | Wang, 1998 [47] | Western Pacific | Shanxi/China | 1997-1999 | Coal miners (a)  Urban children (b) | NR* | | ELISA to detect IgM and IgG | *L. pneumophila* |
| 47 | Wang, 1994 [48] | Western Pacific | Dalian/China | 1992-1993 | Hospital patients (a)  Blood donors (b) |  | | Microagglutination test | *L. pneumophila* sg1,6,8  *L. longbeachae*  *L. micdadei* |
| 48 | Wang, 1990 [49] | Western Pacific | Wuhu, Nanjing/China | Mar 1986-Mar 1987 | General population | NR* | | Microagglutination test | *L. pneumophila* sg1–8 |
| 49 | Yamashiro, 1994 [50] | Western Pacific | Nishihara/Japan | 1990-1993 | Hospital patients (105 cases) | (9) | (1) | IFA | *L. pneumophila* sg1–5  *L. bozemanae*  *L. dumoffii* |
| 50 | Yoon, 2013 [51] | Western Pacific | Seoul, Korea | 2011-2012 | Residential population | (10) | (7) | IFA; ≥1:128 | *L. pneumophila* sg1–6  *L. bozemanae*  *L. gormannii*  *L. longbeachae*  *L. feeleii*  *L. dumoffii*  *L. busanenesis*  *L. anisa* |
| 51 | Javed, 2010 [52] | South East Asian | New Delhi, India | May 2005-Jan 2008 | 1. Control group (hospital and laboratory staff) 2. Paediatric (27) and adult (86) hospital patients | 33 | 11 | Three commercial ELISA commercial kits (EUROIMMUN Medizinisch Labordiagnostika AG, Netherland) to detect IgG, IgM and IgA | *L. pneumophila* sg1–7 |
|  |  |  |  |  |  | 75 | 38 |  |  |
| 52 | Wahala, 2000 [53] | South East Asian | Sri Lanka | NR* | Hotel workers | NR* | | IFA (Scimedex Corporation, USA) ≥1:256 | *L. pneumophila* |
| 53 | Estrich, 2017 [54] | The Americas | United States | 2002-2012  (NB data not collected in 2008, 2010, 2011) | Volunteer U.S. dental practitioners attending the 2002-2012 Health Screening Programs at the American Dental Association’s annual meeting | 3189 | 2148 | ELISA (Wampole Legionella ELISA II Test System, Zeus Scientific); ≥1:256 | *L. pneumophila* sg1–6 |
| 54 | Lobos, 1994 [55] | The Americas | Santiago, Chile | 1991 | Residential population ≤ 20 years (children and adolescents) | NR* | | IFA kit (Organon Teknika, USA);  ≥1:64 | *L. pneumophila* sg1–6 |
| 55 | Lobos, 1993 [56] | The Americas | Santiago, Chile | NR* | Residential adult population 20-80 years | 67 | 33 | IFA kit (Organon Teknika, USA);  ≥1:64 | *L. pneumophila* sg1–6 |
| 56 | Nagalingam, 2005 [57] | The Americas | Trinidad and Tobago | Oct 2002-Oct 2003 | Hospital patients from four hospitals between the ages of 5-70 years | (19) | (20) | IFA kit (Sigma Diagnostics kit of *L. pneumophila* for IgG/IgM and IgA) | *L. pneumophila* sg1–7 |
| 57 | Nichol, 1991 [58] | The Americas | Minneapolis/United States | Aug-Sep 1988 | Non-acutely ill outpatients | 388 | 8 | IFA to detect IgM, IgG and IgA; ≥1:128 | *L. pneumophila* sg1-6 |

NR* = not reported

ELISA = enzyme-linked immunosorbent assay

IFA = immunofluorescence assay

**References**

1. Borella, P. *et al*. Prevalence of anti-legionella antibodies among Italian hospital workers. *J. Hosp Infect.* **69,** 148-155 (2008).

2. Bosca, A., Birengel, S., Kurt, H. & Tekeli, E. Legionella pneumophila as a causative agent of community acquired pneumonia and the efficacy of macrolide antibiotics in the empirical treatment. *Mikrobiyol Bul*. **32,** 209-218 (1998).

3. Boshuizen, H.C. *et al*. Subclinical Legionella infection in workers near the source of a large outbreak of Legionnaires disease. *J. Infect Dis*. **184,** 515-518 (2001).

4. Casal, M., Linares, S.M., Martinez, N.J. & Solis, C.F. Detection of Legionella pneumophila-specific antibody by indirect immunofluorescence assay. *Acta Microbiol Imm H.* **39,** 55-59 (1992).

5. Coniglio, M.A., Pignato, S. & Giammanco, G. Prevalence of antibodies against Legionella spp. in HIV-infected subjects and blood donors. *J. Infect*. **59,** 423-425 (2009).

6. Daniau, C., Wallet, F. & Cabanes, P.A. Seroprevalence survey of antibodies against Legionella pneumophila among exposed and nonexposed personnel at industrial sites. *Environn Risqus Sante*. **9,** 209-216 (2010).

7. Darelid, J. *et al*. Legionella pneumophila serogroup 1 antibody kinetics in patients with Legionnaires' disease: implications for serological diagnosis. *Scand J Infect Dis*. **35,** 15-20 (2003).

8. de Ory, F. & Minguito, T. Comparison of five commercial assays for the detection of Legionella pneumophila antigens in urine. *Enferm Infecc Microbiol Clin*. **27,** 81-84 (2009).

9. Gjenero-Margan, I., Vidovic, R. & Drazenovic, V. Pityriasis rosea gibert: Detection ofLegionella micdadei antibodies in patients. *Eur J Epidemiol*. **11,** 459-462 (1995).

10. Haraldsson, Á., Rechnitzer, C., Friis-Møller, A. & Briem, H. Prevalence of IgM antibodies to nine Legionella species in Icelandic children. *Scand J Infect Dis.* **22,** 445-449 (1990).

11. Heudorf, U., Hentschel, W., Hoffmann, M., Lück, C. & Schubert, R. Legionellas in domestic warm water--effects on the health of residents. *Gesundheitswesen (Germany))*. **63,** 326-334 (2001).

12. Kevorkyan, A. *et al*. Legionella pneumophila antibodies in serum samples from medical and dental personnel: a seroepidemiological survey. *Biotech Biotech Eq*. **31,** 588-593 (2017).

13. Napoli, C., Tato D., Latta, R. & Montagna M.T. Assessment of occupational risk of Legionella spp. infection among dental health-care personnel. *Ig Sanita Pubbl*. **63,** 683-689 (2007).

14. Neubauer, B., Tetzlaff, K., Langfeldt, N. & Mutzbauer, T. Occurance of bacteria pathogenic to man in different types of diving apparatuses. *Int Marit Health*. **50,** 29-37 (1999).

15. Ngeh, J. & Goodbourn C. Chlamydia pneumoniae, Mycoplasma pneumoniae, and Legionella pneumophila in Elderly Patients With Stroke (C-PEPS, M-PEPS, L-PEPS) A Case-Control Study on the Infectious Burden of Atypical Respiratory Pathogens in Elderly Patients With Acute Cerebrovascular Disease. *Stroke*. **36**, 259-265 (2005).

16. Ongut, G. *et al*. Seroprevalence of antibodies to legionella pneumophila in hemodialysis patients. Transplantation proceedings, Elsevier. 44-46 (2004).

17. Pancer, K., Rabczenko, D. & Stypukowska-Misiurewicz, H. The influence of contamination of a hospital hot-water system with Legionella pneumophila on serum antibody production by staff members. *Indoor Built Environ*. **15,** 105-109 (2006).

18. Pankhurst, C. *et al*. Prevalence of legionella waterline contamination and Legionella pneumophila antibodies in general dental practitioners in London and rural Northern Ireland. *Br Dent J*. **195,** 591-594 (2003).

19. Polat, Y., Ergin, C., Kaleli, I. & Pinar, A. Investigation of Legionella pneumophila seropositivity in the professional long distance drivers as a risky occupation. *Mikrobiyol Bul*. **41,** 211-217 (2007).

20. Rocha, G., Verissimo, A., Bowker, R., Bornstein, N. & Da Costa, M. Relationship between Legionella spp. and antibody titres at a therapeutic thermal spa in Portugal. *Epidemiol Infect*. **115,** 79-88 (1995).

21. Rudbeck, M., Molbak, K. & Uldum, S. High prevalence of antibodies to Legionella spp. in Danish blood donors. A study in areas with high and average incidence of Legionnaires' disease. *Epidemiol Infect*. **136,** 257-262 (2008).

22. Rudbeck, M., Mølbak, K. & Uldum, S. Dynamics of Legionella antibody levels during 1 year in a healthy population. *Epidemiol Infect*. **137,** 1013-1018 (2009).

23. Rudbeck, M., Viskum, S., Molbak, K. & Uldum, S.A. Legionella antibodies in a Danish hospital staff with known occupational exposure. *J. Environ Public Health*. **2009,** 1-6 (2009).

24. Sikora, A. *et al*. The occurrence of antibodies against Legionella pneumophila in patients with autoimmune rheumatic diseases. *Pol Arch Med Wewn*. **125,** 749-754 (2015).

25. Sikora, A. *et al*. Prevalence of Legionella antibodies in immunocompromised patients. *Open Med*. **8,** 208-212 (2013).

26. Valciņa, O. *et al*. Legionella pneumophila seropositivity-associated factors in Latvian blood donors. *Int J Environ Res Public Health*. **13,** 58 (2015).

27. Wedege, E. *et al*. Seroepidemiological study after a long-distance industrial outbreak of legionnaires' disease. *Clin Vaccine Immunol*. **16,** 528-534 (2009).

28. Alavi, S.M. *et al*. Seroprevalence of Legionella pneumophila in admitted patients with pneumonia in training hospitals, Ahvaz, Iran (2007-2008). *Pak J Med Sci*. **25,** 811-816 (2009).

29. Lieberman, D. *et al*. Serological evidence of Legionella species infection in acute exacerbation of COPD. *Eur Respir J*. **19,** 392-397 (2002).

30. Razavi, S.M., Ziaee, H., Mokhtari-Azad, T. *et al*. Surveying respiratory infections among Iranian Hajj pilgrims. *Iran J Clin Infect. Dis*. **2,** 67-70 (2007).

31. Bartie, C. & Klugman, K. Exposures to Legionella pneumophila and Chlamydia pneumoniae in South African Mine Workers. *Int J Occup Environ Health*. **3,** 120-127 (1997).

32. Phakkey, A., Lindqvist, K., Omland, T. & Berdal, B.P. Legionella antibodies in human and animal populations in Kenya. *APMIS*. **98,** 43-49 (1990).

33. Bell, J.C. *et al*. Legionellosis linked with a hotel car park–how many were infected? *Epidemiol Infect.* **116,** 185-192 (1996).

34. Heng, B., Goh, K., Ng, D. & Ling, A. Surveillance of legionellosis and Legionella bacteria in the built environment in Singapore. *Ann Acad Med Singapore*. **26,** 557-565 (1997).

35. Hsu, C.M. *et al*. Prevalence of Legionella pneumophila infection in children and its role in pediatric community-acquired atypical pneumonia. *Zhonghua Minguo xiao er ke yi xue hui*. **37,** 188-192 (1996).

36. Jiang H, Shao Z, Li J, Zou M. Inspection on Legionella pollution and the health impact of workers in public places of central air conditioning systems in Dalian City. *Wei Sheng Yan Jiu*. **38,** 76-77 (2009).

37. Lee, H.K. *et al*. Prevalence of antibodies in response to Legionella species, analysis of a healthy population from Jeollanam-do Province, Korea. *J Microbiol*. **46,** 160-164 (2008).

38. McGrath, T., Douglas, K. & McGarry, P. Report on Investigation of Background Levels of Antibodies to *Legionella*. (Department of Industrial Relations, Queensland Government, 2006).

39. Mineshita, M., Nakamori, Y., Seida, Y. & Hiwatashi, S. Legionella pneumonia due to exposure to 24-hour bath water contaminated by Legionella pneumophila serogroup-5. *Internal Med*. **44,** 662-665 (2005).

40. Morimoto, T. Serum antibodies to Legionella pneumophila by indirect immunofluorescent antibody test in 269 hemodialysis patients and 353 healthy subjects. *Rinsho byori*. **39,** 71-75 (1991).

41. Pan, T., Yea, H., Huang, H., Lee, C. & Horng, C. Legionella pneumophila infection in Taiwan: a preliminary report. *J Formosan Med Assoc*. **95,** 536-539 (1996).

42. Peng, X., Pei, H. & Li, X. *et al*. Studies on Legionella-contamination to the air-conditioning cooling towers in big hotels and on its seroprevalence in the related populations in Beijing. *Zhonghua liu xing bing xue za zhi= Zhonghua liuxingbingxue zazhi*. **21,** 289-291 (2000).

43. Queensland Health. *Prevalence of antibodies to six Legionella species in commercial nursery workers*. Brisbane, Australia. (1992).

44. Sakamoto, R. *et al*. Is driving a car a risk for Legionnaires' disease? *Epidemiol Infect*. **137,** 1615-1622 (2009).

45. Sun, H. *et al*. Cooling Towers contribute to the high seroprevalence of Legionella pneumophila antibody among hotel workers. *J. Public Health*. **20,** 425-430 (2012).

46. Tay, S.T., Lakhbeer Singh, H., Ramasame, S.D. & Vadivelu J. Detection of IgM antibodies against Legionella pneumophila serogroup 1 in Malaysian blood donors and patients with respiratory illnesses: evaluation of enzyme-linked immunosorbent assay and indirect immunofluorescence assay. *Jpn J Infect Dis*. **62,** 409-410 (2009).

47. Wang, S., Wang, J. & Zhang, Z. Epidemiological studies on Legionella infection in Taiyuan area of Shanxi Province. *Zhonghua yu fang yi xue za zhi*. **32,** 208-210 (1998).

48. Wang, Y., Lei, Z. & Ju, C. Comparative study on Legionella infection in different groups of population in Dalian area. *Zhonghua liu xing bing xue za zhi= Zhonghua liuxingbingxue zazhi*. **15,** 271-274 (1994).

49. Wang, N. Study on seasonality of 1-8 serotypes legionella pneumophila infections. *Zhonghua liu xing bing xue za zhi= Zhonghua liuxingbingxue zazhi.* **11,** 68-71 (1990).

50. Yamashiro, Y. *et al*. Serodiagnosis of Legionella pneumonia--data of our laboratory in the recent 3 years. *Kansenshogaku zasshi*. **68,** 1256-1263 (1994).

51. Yoon, Y.T. *et al*. An Epidemiological Survey on Serological Diagnosis of Legionella Infection in Seoul, Korea. *J. Bacteriology Virol*. **43,** 140-144 (2013).

52. Javed, S. *et al*. Sero diagnosis of Legionella infection in community acquired pneumonia. *Indian J Med Res.* **131,** 92-96 (2010).

53. Wahala, W. & Wickramasinghe, R. First isolation of Legionella pneumophila in Sri Lanka. *Ceylon Med J*. **45,** 171-172 (2000).

54. Estrich, C.G., Gruninger, S.E. & Lipman, R.D. Rates and predictors of exposure to Legionella pneumophila in the United States among dental practitioners: 2002 through 2012. *J. Am Dent Assoc*. **148,** 164-171 (2017).

55. Lobos, T. *et al*. Seroprevalencia de Legionella pneumophila en Santiago: evidencias de exposición durante la infancia. *Rev Chil Pediatr*. **65,** 197-200 (1994).

56. Lobos, T. *et al*. Seroprevalence of Legionella pneumophila infection in healthy, adults from Santiago, Chile. *Rev Med Chile*. **121,** 1123-1127 (1993).

57. Nagalingam, N.A. *et al*. Seroprevalence of Legionella pneumophila in pneumonia patients in four major hospitals in Trinidad and Tobago. *West Indian Med J*. **54,** 375-378 (2005).

58. Nichol, K.L., Parenti, C.M. & Johnson, J.E. High prevalence of positive antibodies to Legionella pneumophila among outpatients. *Chest*. **100,** 663-666 (1991).
